# Dystrophin restoration therapy improves both the reduced excitability and the force drop induced by lengthening contractions in dystrophic mdx skeletal muscle

**DOI:** 10.1186/s13395-016-0096-4

**Published:** 2016-07-20

**Authors:** Pauline Roy, Fredérique Rau, Julien Ochala, Julien Messéant, Bodvael Fraysse, Jeanne Lainé, Onnik Agbulut, Gillian Butler-Browne, Denis Furling, Arnaud Ferry

**Affiliations:** Groupe Hospitalier Pitié Salpêtrière, Centre de Recherche en Myologie, CNRS, Inserm, UPMC Univ Paris 06, Sorbonne Universités, Paris, F-75013 France; Centre of Human and Aerospace Physiological Sciences, King’s College London, Guy’s Campus, SE3 8TL London, UK; Biological Adaptation and Ageing, UMR CNRS 8256, Institut de Biologie Paris-Seine (IBPS), UPMC Univ Paris 06, Sorbonne Universités, Paris, F-75005 France; Sorbonne Paris Cité, Université Paris Descartes, Paris, F-75006 France; Groupe Hospitalier Pitié-Salpétrière, Institut de Myologie, F-75013 Paris, France

## Abstract

**Background:**

The greater susceptibility to contraction-induced skeletal muscle injury (fragility) is an important dystrophic feature and tool for testing preclinic dystrophin-based therapies for Duchenne muscular dystrophy. However, how these therapies reduce the muscle fragility is not clear.

**Methods:**

To address this question, we first determined the event(s) of the excitation-contraction cycle which is/are altered following lengthening (eccentric) contractions in the mdx muscle.

**Results:**

We found that the immediate force drop following lengthening contractions, a widely used measure of muscle fragility, was associated with reduced muscle excitability. Moreover, the force drop can be mimicked by an experimental reduction in muscle excitation of uninjured muscle. Furthermore, the force drop was not related to major neuromuscular transmission failure, excitation-contraction uncoupling, and myofibrillar impairment. Secondly, and importantly, the re-expression of functional truncated dystrophin in the muscle of mdx mice using an exon skipping strategy partially prevented the reductions in both force drop and muscle excitability following lengthening contractions.

**Conclusion:**

We demonstrated for the first time that (i) the increased susceptibility to contraction-induced muscle injury in mdx mice is mainly attributable to reduced muscle excitability; (ii) dystrophin-based therapy improves fragility of the dystrophic skeletal muscle by preventing reduction in muscle excitability.

## Background

Duchenne muscular dystrophy (DMD) is a severe disorder affecting both skeletal and cardiac muscles resulting from dystrophin deficiency. Dystrophin, a subsarcolemmal protein encoded by *Dmd*, is thought to play a role in sarcolemma stability, localization, and function of different proteins that trigger damage process when absent [1, 2]. In the case of dystrophin deficiency, the skeletal muscle is more fragile, i.e., more susceptible to damage caused by high-force contractions both in situ and in vitro, such as produced during lengthening contractions, also known as eccentric contractions [3–6]. Lengthening contractions occur when the muscle acts as a brake, to slow down movement consequently. Cycles of muscle injury and incomplete recovery occur and contribute to progressive muscle weakness and loss in DMD patients.

The immediate force drop following lengthening contractions is a widely used measure of the magnitude of muscle damage caused by contraction in dystrophin-deficient mdx mice [7], the most widely used mouse model for DMD. Thus, it is an important tool for testing preclinic dystrophin-based therapies for DMD. Interestingly, dystrophin-based therapies reduce the extent of the immediate force drop following lengthening contractions in mdx mice [8–15]. However, it remains unclear how these therapies reduce the susceptibility to lengthening contraction-induced injury in mdx mice.

The immediate force drop following repetitive lengthening contractions observed in mdx muscles (>50 % after less than 10 contractions) is theoretically related to changes in the cascade of events responsible for muscle excitation and contraction. It results from a failure in neuromuscular transmission, reduced muscle excitability, impaired calcium release and uptake in the sarcoplasmic reticulum, and/or contractile impairment. However, the precise events of the cycle of excitation-contraction responsible for the immediate force drop following lengthening contractions are still being clarified. A recent work postulates that neuromuscular transmission failure contributes to the greater force drop following lengthening contractions in mdx mice [16, 17]. Another recent study suggests that reduced muscle excitability is a major mechanism of the greater force drop in mdx mice [18]. Moreover, it was previously proposed that the greater force drop in mdx mice results from myofibrillar impairment due to structural damage [19, 20]. Overall, these finding revealed that the mechanisms of the greater force drop following lengthening contractions in mdx are variable and somewhat contradictory.

The aims of the present study were to (i) determine the altered event(s) of the excitation contraction cycle leading to the immediate force drop following lengthening contractions in mdx mice and (ii) demonstrate that dystrophin restoration-based therapy improves the defective event(s) of the excitation-contraction cycle. In the present study, we studied neuromuscular transmission, muscle excitability, excitation-contraction coupling, and myofibrillar function in the hindlimb skeletal muscle of mdx mice, by combining a broad range of biophysical and biological assays. The restoration of dystrophin expression in mdx mice was performed by *Dmd* exon 23 skipping strategy [10].

## Methods

### Animals

All procedures were performed in accordance with the National and European legislations. Mdx mice (mdx, C57BL/10ScSc-DMD^mdx^/J) and sex- and age-matched wild-type control mice (C57) (healthy) were used at 3–6 months of age.

### Whole muscle force production in response to electrical stimulation

Maximal tetanic isometric force and susceptibility to contraction-induced injury (see below) were evaluated by measuring the in situ tibialis anterior (TA) and extensor digitorum longus (EDL) muscle contraction in response to nerve stimulation, as described previously [21, 22]. Mice were anesthetized using pentobarbital (60 mg/kg ip). Body temperature was maintained at 37 °C using radiant heat. The knee and foot were fixed with pins and clamps, and the distal tendon of the muscle was attached to a lever arm of a servomotor system (305B, Dual-Mode Lever, Aurora Scientific, Aurora, Canada) using a silk ligature. The sciatic nerve was proximally crushed and distally stimulated by a bipolar silver electrode using supramaximal square wave pulses of 0.1-ms duration (10 V). We measured the absolute maximal force that was generated during isometric tetanic contractions in response to electrical stimulation (125 Hz, 500 ms). Absolute maximal force was determined at L0 (length at which maximal tension was obtained during the tetanus). Absolute maximal force was normalized to the muscle weight as an estimate of specific maximal force (absolute maximal force/muscle weight). In some case, submaximal force in response to nerve stimulation (25 Hz, 500 ms) was also measured to calculate the 25 Hz/125 Hz force ratio.

Susceptibility to contraction-induced injury was estimated from the force drop resulting from lengthening contraction-induced injury. The sciatic nerve was stimulated for 700 ms (frequency of 125 Hz). A maximal isometric contraction of the TA muscle was initiated during the first 500 ms. Then, muscle lengthening (10 % L0) at a velocity of 5.5 mm/s (0.85 fiber length/s) was imposed during the last 200 ms. Nine lengthening contractions of the muscle were performed, each separated by a 60-s rest period. All contractions were made at an initial length L0. Maximal isometric force was measured 60 s after each lengthening contraction and expressed as a percentage of the initial maximal force.

In some cases, before and after the last lengthening contraction, stimulating electrodes were applied directly on the muscle, which was injected, or not with tubocurarine, a neuromuscular transmission blocker. Direct muscle stimulation was performed in order to evaluate neuromuscular transmission (without tubocurarine injection) and muscle excitability (with tubocurarine injection). Comparisons between nerve (10 V) and muscle (80 V) stimulations were made to evaluate nerve-muscle communication [23]. A lower force produced in response to nerve stimulation versus muscle stimulation was indicative of a defect in neuromuscular transmission. We increased the muscle stimulation strength (5–95 V) to determine the voltage strength needs to produce 50 % of maximal force, an index of muscle excitability.

Data was acquired with a sampling rate of 100 kHz (Powerlab 4/25, ADInstrument, Oxford, UK). After contractile measurements, the animals were killed by cervical dislocation and muscles were weighed.

### Muscle action potential

Electromyography was performed with anesthetized mice (pentobarbital, 60 mg/kg ip) in order to complete the evaluation of neuromuscular transmission and muscle excitability (see above). For compound muscle action potential (CMAP) recordings, two monopolar needle electrodes were inserted into the belly of the TA muscle. The recording (cathode) and the reference (anode) electrodes were inserted, respectively, into the 1/3 proximal and the distal portion of the muscle. A third monopolar electrode was inserted in the contralateral hindlimb muscle to ground the system. Data was amplified (BioAmp, ADInstrument), acquired with a sampling rate of 100 kHz, and filtered at 5 kHz low pass and 1 Hz high pass (Powerlab 4/25, ADInstrument). Recording electrodes were positioned to achieve maximal CMAP amplitude.

Nerve stimulations (0.1 ms, 10 V, 3 Hz and 10 Hz, stimulation trains for 20 s) were applied to the sciatic nerve, and CMAP was recorded to search for decrementing response to repetitive stimulation, used as a marker of neuromuscular transmission failure [24]. CMAP amplitude was measured peak-to-peak and was expressed as percentage of the first CMAP. CMAP were also recorded during lengthening and isometric contractions, and we calculated the root mean square (RMS) of CMAP, as an index of CMAP amplitude. RMS of each CMAP corresponding to each contraction was then expressed as a percentage of the first contraction, used as a marker of muscle excitability.

### Myofibrillar contractility

To evaluate myofibrillar contractility, skinned fibers were studied as previously described [25]. Immediately after the lengthening contractions, TA muscles were dissected and placed in an ice-cold relaxing solution (in mmol/l: 100 KCl, 20 Imidazole, 7 MgCl2, 2 EGTA, 4 ATP, pH 7.0; 4 °C). Small bundles of ~25–50 fibers were dissected free from the muscle and tied to a glass microcapillary tube at ~110 % resting length. The bundles were then placed in a skinning solution (relax solution containing glycerol; 50:50 *v*/*v*) at 4 °C for 24 h and subsequently treated with a cryoprotectant (sucrose solution) for long-term storage at −80 °C as described earlier [26]. On the day of the experiment, a bundle was desucrosed and single fibers isolated. A fiber segment length of 1 to 2 mm was then left exposed to the relaxing solution between connectors leading to a force transducer (model 400A, Aurora Scientific) and a lever arm system (model 308B, Aurora Scientific). The apparatus was mounted on the stage of an inverted microscope (model IX70; Olympus). While the fiber segment was in relaxing solution, the sarcomere length was set to 2.50 ± 0.05 μm by adjusting the overall segment length. The sarcomere length was controlled during the experiments using a high-speed video analysis system (model 901A HVSL, Aurora Scientific). The fiber segment width, depth, and length between the connectors were measured. Fiber cross-sectional area (CSA) was calculated from the diameter and depth, assuming an elliptical circumference, and was corrected for the 20 % swelling that is known to occur during skinning. At 15 °C, immediately preceding each activation, the fiber segment was immersed for 10–20 s in a solution with a reduced Ca2+-EGTA buffering capacity. This solution is identical to the relaxing solution except that the EGTA concentration is reduced to 0.5 mM, which results in more rapid attainment of steady force during subsequent activation. Maximal isometric force was calculated as the difference between the total force in activating solution (pCa 4.5) and the resting force measured in the same segment while in the relaxing solution. Maximal force was adjusted for fiber CSA and termed specific maximal force (P0). After mechanical measurements, each fiber was placed in urea buffer in a plastic microcentrifuge tube and stored at −80 °C until analysis by gel electrophoresis. The myosin heavy chain (MHC) isoform composition of fibers was determined by 6 % SDS-PAGE. The acrylamide concentration was 4 % (*w*/*v*) in the stacking gel and 6 % in the running gel, and the gel matrix included 30 % glycerol. Sample loads were kept small (equivalent to ~0.05 mm of fiber segment) to improve the resolution of the MHC bands (slow and fast MyHC: types I, IIa, IIx, and IIb). Electrophoresis was performed at 120 V for 24 h with a Tris-glycine electrode buffer (pH 8.3) at 15 °C (SE 600 vertical slab gel unit, Hoefer Scientific Instruments). The gels were silver-stained and subsequently scanned in a soft laser densitometer (molecular dynamics) with a high spatial resolution (50 μm pixel spacing) and 4096 optical density levels. Fibers were either expressing the type IIx or IIb MHC isoforms. As the force measurements were not different between these two types, data were pooled together.

### Muscle fiber ultrastructure

To evaluate fiber ultrastructure integrity immediately after lengthening contractions, electron microscopy was performed on TA muscles first fixed with 2 % PFA, 2 % glutaraldehyde in 0.1 M phosphate buffer (pH 7.4), and then with 2 % OsO4 in 0.1 M phosphate buffer for 1 h at 4 °C. Muscles were then dehydrated at 4 °C in graded acetone including a 1 % uranyl acetate in 70° acetone staining step, before Epon resin embedding. Thin (70 nm) sections were stained with uranyl acetate and lead citrate and observed using a Philips CM120 electron microscope (Philips Electronics NV) and photographed with a digital SIS Morada camera.

### Acetylcholine receptor morphology

Acetylcholine receptors (AChR) staining on isolated muscle fibers was performed as described previously [27]. Briefly, TA muscles were dissected immediately after lengthening contractions and fixed in 4 % PFA/PBS for 30 min and rinsed with PBS, pH 7.5, at room temperature. Isolated muscle fibers were incubated for 15 min with 100 mM glycine in PBS and rinsed in PBS. Samples were permeabilized and blocked in blocking buffer (3 % BSA/5 % goat serum/0.5 % Triton X-100/PBS) for 4 h at room temperature. They were then incubated overnight at 4 °C with α-bungarotoxin (α-BTX) Alexa Fluor® 488 conjugate (Life Technologies, 1/1000) in blocking buffer. Finally, after four 1-h washes in PBS, muscles were flat-mounted in Vectashield (Vector Labs) mounting medium. Confocal images were acquired using Leica SPE confocal microscope with a Plan Apo ×63 NA 1.4 oil objective (HCX; Leica). Confocal software (LAS AF; Leica) was used for acquisition of Z serial images, with a Plan Apo ×63 NA 1.4 oil objective (HCX; Leica). Confocal images presented are single-projected image derived from image stacks. For all imaging, exposure settings were identical between compared samples and genotypes. Quantifications were done as previously [28], using ImageJ software (version 1.46 m). Total neuromuscular junction area was determined by delineating the outside edges of AChR clusters. AChR rich-endplate area per neuromuscular junction corresponds to the occupied area of α-bungarotoxin fluorescent signal.

### Exon skipping-based dystrophin restoration

The restoration of dystrophin expression was performed by *Dmd* exon 23 skipping strategy using optimized U7-small nuclear RNA (snRNA) antisense sequence (U7ex23) previously described [10, 25]. Adeno-associated vectors (AAV1) carrying the U7ex23 constructs were injected in Tibialis anterior TA muscles from mdx mice. Titer for AAV1-U7ex23 was 1.2 × 10^12^ vector genomes (vg) ml^−1^. Briefly, mice were anesthetized (2–4 % isoflurane) and TA muscles of the right hindlimb were injected (40 μl, 5.0 × 10^10^ vg). Control muscle was obtained from the left hind limb injected with saline solution only. Muscles were collected 3 weeks after injection.

### RNA isolation and quantification of *Dmd* Exon 23 skipping

In order to assess the level of exon 23 skipping, RT-PCR analysis was done. Total RNA was isolated from TA muscle samples using Tri Reagent (Sigma) according to the manufacturer’s protocol. One microgram of RNA was reverse transcribed using Enhances Avian Reverse Transcriptase (eAMV™ RT), according to the manufacturer’s instruction (Sigma). Non-skipped and skipped dystrophin transcripts were detected by nested PCR and quantified as previously described [29]. The ratio of exon inclusion/exclusion was quantified with ImageJ software and as a percentage of inclusion/exclusion relative to total intensity of isoform signals.

### Quantitative PCR for dystrophin

The level of dystrophin messenger RNA (mRNA) was assessed by q-PCR analysis using a Lightcycler 480 (Roche). Reactions were performed with SYBR Green kit (Roche) according to the manufacturer’s instructions. PCR cycles were a 15-min denaturation step followed by 50 cycles with 94 °C denaturation for 15 s, 58 °C annealing for 20 s, and 72 °C extension for 20 s. Mouse Rrlp0 mRNA was used as standard. Data were analyzed with the Lightcycler 480 analysis software. Primer sequences: mouse *Dmd forward*: *5′-TGGATCTGACATCTCATCAAGGAC-3′*; *mouse Dmd reverse*: *5′-CCATGCTAGCTACCCTGAGAC-3′*; *mouse Rrlp0 forward*: *5′-GAGGACCTCACTGAGATTCGG-3′*; *mouse Rrlp0 reverse*: *5′-TTCTGAGCTGGCACAGTGAC-3′*.

### Western blot analysis of dystrophin

To confirm the presence of dystrophin protein expression, Western blots were performed. Total protein was extracted from TA muscle samples with lysis buffer (50 mM Tris-HCl pH 8.0, 150 mM NaCl, 1 % Triton, 1 % sodium deoxycholate, 0.1 % SDS, and Complete Protease inhibitor cocktail (Roche)) and quantified using BCA Protein Assay Kit (Thermo Scientific Pierce). After a denaturation step for 5 min at 95 °C, 50 μg of total protein extract was loaded in Novex 4–12 % Bis-Tris protein gels (Life Technologies) and transferred to nitrocellulose membrane. Blots were blocked for 1 h with 10 % non-fat milk in Tris-buffered saline. Dystrophin and alpha-actinin proteins were detected by probing the membrane with 1:100 dilution of monoclonal NCL-DYS-1 primary antibody (Novocastra) and 1:1000 of monoclonal anti-alpha-actinin primary antibody (Sigma), respectively. An incubation with 1:5000 of sheep anti-mouse secondary antibody (horseradish peroxidase conjugated) allowed visualisation using Substrat HRP Immobilon Western (Millipore). Band intensities were analyzed using ImageJ software.

### Statistical analysis

Groups were statistically compared using one- or two-way analysis of variance (lengthening contractions, genotype × lengthening contractions…) and Student’s *t* test. If necessary, subsequent Bonferroni post hoc test was also performed. Values are means ± SEM.

## Results

### Force dramatically drops after lengthening contractions in mdx but not C57 mice

**Fig. 1:**
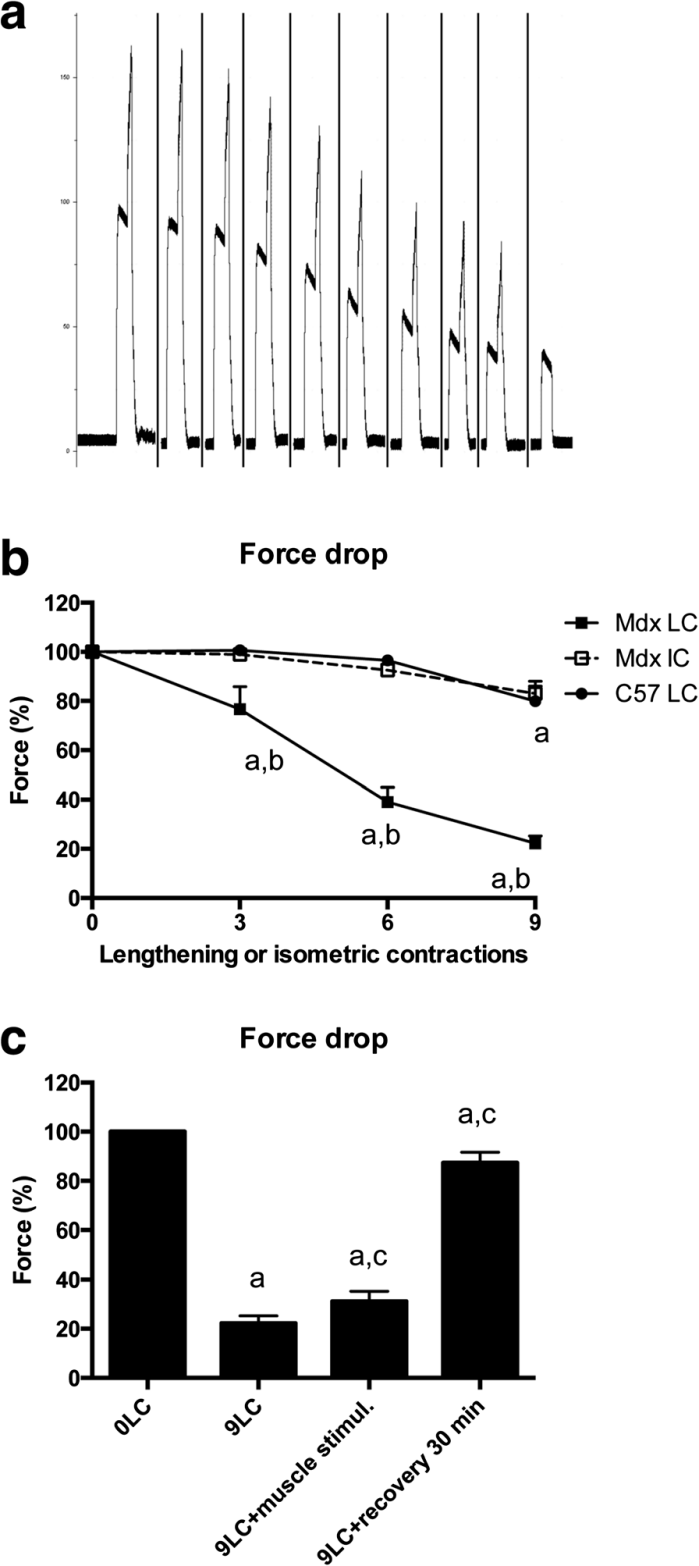
Susceptibility to contraction-induced muscle injury, i.e., force drop following lengthening contractions in TA muscle from mdx mice and neuromuscular transmission. **a** Example of force traces during lengthening contractions in mdx mice. The 10th contraction was isometric. **b** Force drop following lengthening and isometric contractions in mdx and C57 mice. **c** Force drop following nine lengthening contractions in mdx mice, recovery, and direct muscle stimulation with high voltage. *Mdx LC* lengthening contractions in mdx mice, *Mdx IC* isometric contractions in mdx mice, *C57 LC* lengthening contractions in C57 mice, *0LC* before lengthening contractions, *9LC* after nine lengthening contractions, *9LC + muscle stimul* stimulating electrodes were located on the muscle after the ninth contractions, *9LC + recovery 30 min* force was measured 30 min after the ninth lengthening contractions, *a* significantly different from before lengthening contractions (*p* < 0.05), *b* significant difference between strains (*p* < 0.05), *c* significant difference from 9LC (*p* < 0.05). *n* = 8–21 per group

By analyzing in situ TA isometric muscle force production in response to tetanic nerve stimulation (125 Hz, 500 ms), we observed an immediate force drop following the third, sixth, and ninth lengthening contractions in mdx mice (*p* < 0.05) (Fig. 1a, b). There was no major force drop in C57 mice (Fig. 1b). In contrast, there was no force drop following isometric contractions in mdx mice (*p* > 0.05), indicating that the force drop was related to the muscle lengthening, but not fatigue, and may be due to the higher force production which occurs during lengthening versus isometric contractions (+65.9 ± 3.6 % in mdx mice and +68.9 ± 4.0 % in C57 mice). It should be noted that maximal force was not fully recovered even after 30 min of recovery (Fig. 1c).

### Is neuromuscular transmission impaired?

To determine whether neuromuscular transmission failure contributes to force drop, we performed electrical TA muscle stimulation that can directly initiate muscle action potentials, without the need of neuromuscular transmission [30, 31]. Stimulating electrodes were positioned on the midbelly of the muscle, and the muscle was stimulated with a high strength voltage (80 V). Under basal conditions (before lengthening contractions), nerve and muscle stimulations produced the same maximal force (data not shown). We found that direct muscle stimulation with a high strength voltage did not markedly improve maximal force production after the nine lengthening contractions in mdx mice (Fig. 1b).

**Fig. 2:**
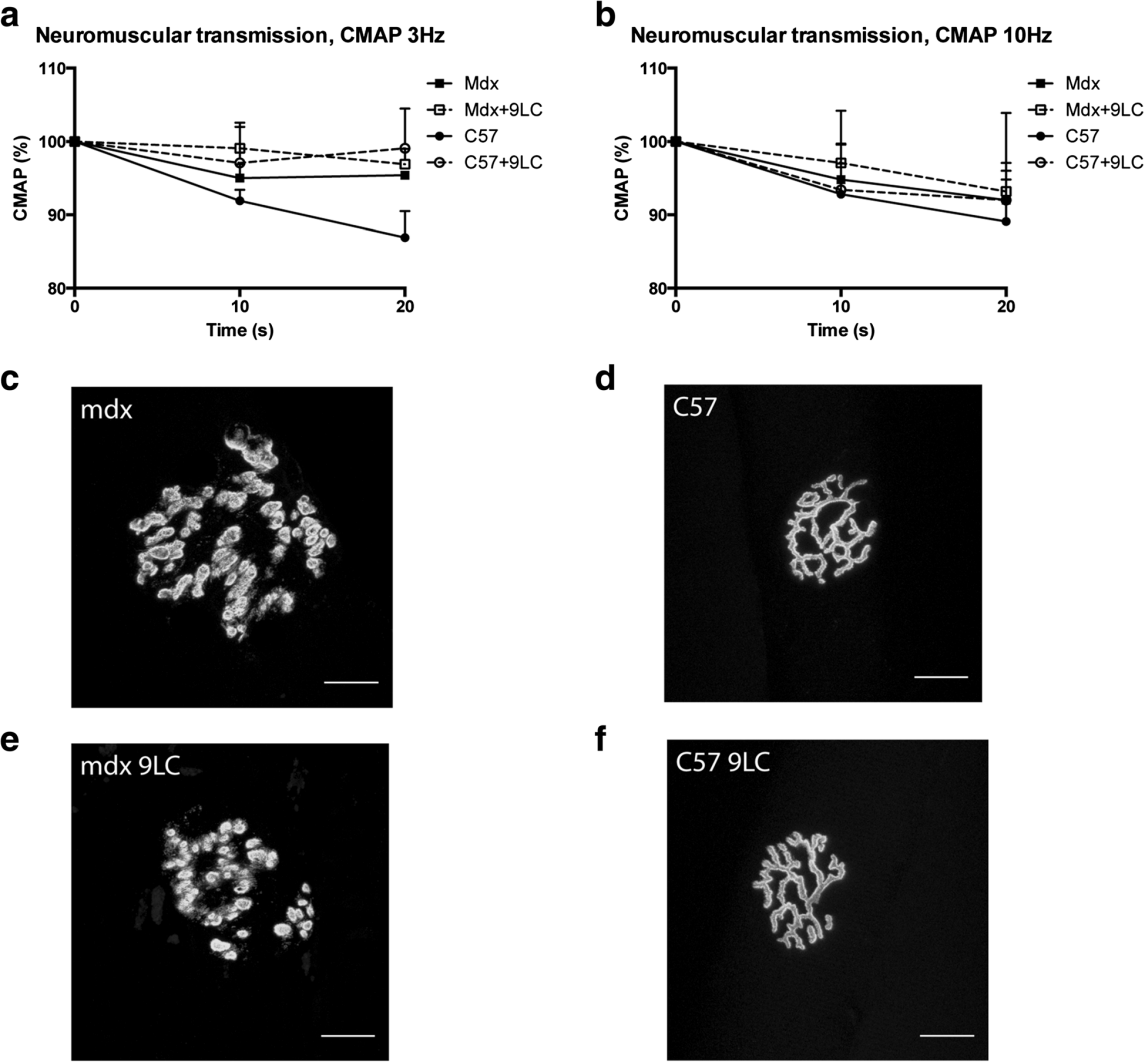
Neuromuscular transmission and neuromuscular junction morphology following lengthening contractions in the TA muscle from mdx mice. **a** Compound muscle action potential (CMAP) (amplitude) in response to 3 Hz nerve stimulation. **b** CMAP (amplitude) in response to 10 Hz nerve stimulation. **c**–**f** representative images of neuromuscular junction morphology using α-bungarotoxin staining. Bar = 20 μm. *CMAP* compound muscle action potential, *Mdx + 9LC* after nine lengthening contractions, *Mdx LC* lengthening contractions in mdx mice, *Mdx and C57* before lengthening contractions. *n* = 6–13 per group for **a** and **b**

Moreover, we performed electromyographic measurements in order to measure CMAP decrement following repetitive nerve stimulation (0.1 ms, 3 and 10 Hz, 20 s). CMAP (amplitude) decrement following 3 or 10 Hz stimulation was higher in the case of neuromuscular transmission failure [24]. We found no 3 and 10 Hz CMAP decrement differences between mdx and C57 mice before and after the nine lengthening contractions (Fig. 2a, b).

To determine whether lengthening contractions cause immediate neuromuscular junction structural changes in mdx mice as previously described [17], fibers from the TA muscle were stained with α-BTX to label acetylcholine receptor (AChR) clusters. Subsequently, z-serial images were collected with a confocal microscope and collapsed into single images. Neuromuscular junctions of mdx mice were severely fragmented before lengthening contractions (Fig. 2c). Indeed, postsynatic network was discontinuous and isolated AChR clusters were observed, indicating a severe dismantlement of neuromuscular junctions. In contrast, AChR clusters exhibited characteristic pretzel-like morphology, with complex continuous branched network, in C57 mice (Fig. 2d). Importantly, the nine lengthening contractions did not appear to markedly affect neuromuscular junction morphology, in both mdx and C57 (Fig. 2e, f). We measured the total neuromuscular junction area, defined by the area contained within a boundary encompassing the outermost edges of the neuromuscular junction, as revealed by α-BTX label. We found a decrease in total neuromuscular junction area in mdx mice following the nine lengthening contractions (*p* < 0.05) but not in C57 mice (Table 1). Since the neuromuscular junction area contained within the outside boundary does not really reflected the occupied area of AChR clusters, we also measured the AChR-rich endplate area per neuromuscular junction corresponding to the α-BTX-labeled area per synapse. Accordingly, the occupied AChR cluster area was also reduced following the nine lengthening contractions in mdx mice (*p* < 0.05) (Table 1). However, the complexity within neuromuscular junction (calculated as (AChR-rich endplate area/total neuromuscular junction area)*100) was unchanged following the nine lengthening contractions in mdx mice (Table 1). Moreover, we found no effect of the nine lengthening contractions on the number of fragments and the area of each AChR fragment in mdx mice (Table 1).

**Table 1:** Morphology parameters of neuromuscular junction following lengthening contractions in mdx mice

	Mdx	Mdx + 9LC	C57	C57 + 9LC
Total NMJ area (μm^2^)	2067.6 ± 145.7	1678.9 ± 83.0^a^	1025.2 ± 69.0	1065.8 ± 44.3
AChR area/NMJ (μm^2^)	1005.2 ± 68.7	775.3 ± 30.3^a^	512.6 ± 29.3	590.1 ± 33.0
Complexity within NMJ (%)	47.6 ± 2.2	48.7 ± 1.3	50.7 ± 1.7	56.0 ± 2.3
AChR fragment number	31.3 ± 2.7	29.1 ± 1.7	3.1 ± 0.7	3.5 ± 0.4
AChR fragment area (μm^2^)	75.6 ± 29.1	34.7 ± 4.3	276.6 ± 60.7	260.5 ± 34.7

*n* > 30 (C57) or >60 (mdx) per group (from 3 C57 mice and 4 mdx mice)

*NMJ* neuromuscular junction, *AChR* acetylcholine receptor, *Mdx or C57* before nine lengthening contractions, *Mdx + 9LC* after nine lengthening contractions, *C57 + 9LC* after nine lengthening contractions

^a^Significantly different from before nine lengthening contractions (*p* < 0.05)

Together, these results indicate that neuromuscular transmission failure was not a major mechanism of the force drop following lengthening contractions in mdx mice.

### Is muscle excitability depressed?

**Fig. 3:**
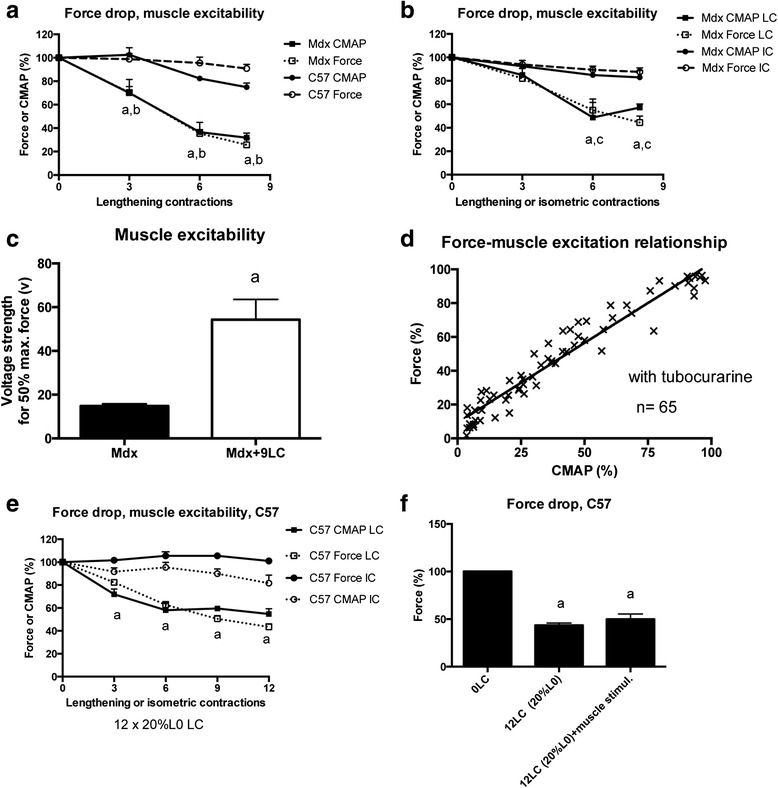
Muscle excitability following lengthening contractions in the TA muscle from mdx mice. Compound muscle action potential (*CMAP*) was measured in response to nerve (**a**, **b**, and **d**) or muscle stimulation (**c**). **a** CMAP (root mean square, RMS) in response to nerve stimulation recorded during lengthening contractions in mdx mice. The force drop was also shown. **b** CMAP (RMS) in response to nerve stimulation recorded during lengthening or isometric contractions in mdx mice. The force drop was also shown. **c** Voltage strength (V) needs to obtain 50 % of maximal force in response to muscle stimulation when neuromuscular transmission was inhibited by tubocurarine in mdx mice. **d** Force and CMAP (RMS) relationship obtained when CMAP in response to nerve stimulation was experimentally reduced by tubocurarine, in the mdx muscle at the basal state (before lengthening contractions). **e** CMAP (root mean square, RMS) in response to nerve stimulation recorded during lengthening and isometric contractions in C57 mice. The force drop was also shown and was induced by a severe lengthening contraction protocol (12 × 20 % L0). **f** Force drop following nine lengthening contractions in C57 mice and direct muscle stimulation with high voltage (80 V). *CMAP* compound muscle action potential, *Mdx + 9LC* after nine lengthening contractions in mdx mice, *Mdx LC* lengthening contractions in mdx mice, *Mdx IC* isometric contractions in mdx mice, *12LC (20 % L0)* 12 × 20 % lengthening contractions were performed in C57 mice, *12LC(20 % L0) + muscle stimul* stimulating electrodes were located on the muscle after 12 × 20 % L0 lengthening contractions in C57 mice, *C57 LC* 12 × 20 % L0 lengthening contractions in C57 mice, *C57 IC* 12 isometric contractions in C57 mice, *a* significantly different from before lengthening contractions (*p* < 0.05), *b* significant difference between strains (*p* < 0.05), *c* significant difference from IC (*p* < 0.05). *n* = 6–13 per group

A decreased generation and propagation of muscle action potential, i.e., reduced muscle excitability, could contribute to the force drop following lengthening contractions. To test this hypothesis, we simultaneously measured maximal force and CMAP (RMS) during lengthening contractions. We found that CMAP in response to nerve stimulation (0.1 ms, 500 ms, 125 Hz) decreased following lengthening contractions in mdx mice (*p* < 0.05), similar to maximal force, but not in C57 mice (Fig. 3a). In contrast, CMAP did not decrease in mdx mice following isometric contractions (Fig. 3b).

Next, we determined the necessary muscle stimulation strength (in V) for 50 % of maximal force production when the muscle is directly activated, i.e., not excited via neuromuscular transmission. TA muscles were injected with tubocurarine (15 μl at 0.07 mg/ml), a neuromuscular transmission blocker. The stimulating electrodes were positioned on the muscle surface. We found that the voltage needed to elicit 50 % of maximal force markedly increased following the nine lengthening contractions (Fig. 3c) (*p* < 0.05), indicating an increased threshold for action potential generation, confirming reduced excitability.

Then, we determined the relationship between CMAP (RMS) and force in intact mdx muscles (that did not performed lengthening contractions) to determine whether an experimental reduction in CMAP caused a proportional decreases in force. TA muscles from mdx mice were injected with various doses of tubocurarine (15 μl at 0.007–0.07 mg/ml) in order to pharmacologically reduced CMAP, and force was measured 5 to 15 min after. We found that absolute maximal force decreased proportionally with CMAP (Fig. 3d). In fact, linear regression analysis revealed a strong correlation between CMAP and absolute maximal force (*r*^2^ = 0.93) (*p* < 0.0001). Since the slope of the regression line was ~1 (0.94 ± 0.03), a given reduction in CMAP caused a similar decrease in force. Therefore, the force drop following lengthening contractions could be mimicked by an experimental reduction in CMAP, i.e., muscle excitation. Together, these results indicate that the reduced muscle excitability contributes to the force drop following lengthening contractions in mdx mice.

To determine whether reduced muscle excitability was also reduced in C57 mice when the force drop is important, they performed 12 × 20 % L0 lengthening contractions, a more severe lengthening contraction protocol than the protocol used for mdx mice (9 × 10 % L0 lengthening contractions). We found that 12 × 20 % L0 lengthening contractions induced a marked force drop (up to −67 %) in C57 mice (Fig. 3e, f). Electromyography analysis indicated that CMAP was also decreased following 12 × 20 % L0 lengthening contractions in C57 mice (*p* < 0.05), but not isometric contractions (Fig. 3e). Moreover, muscle stimulation with high strength current (80 V) did not markedly reduce the force drop following lengthening contractions in C57 mice (Fig. 3f), indicating that the reduced CMAP was independent from neuromuscular transmission failure. Together, these results suggest that reduced muscle excitability is also a mechanism of the force drop following lengthening contractions in C57 mice.

**Fig. 4:**
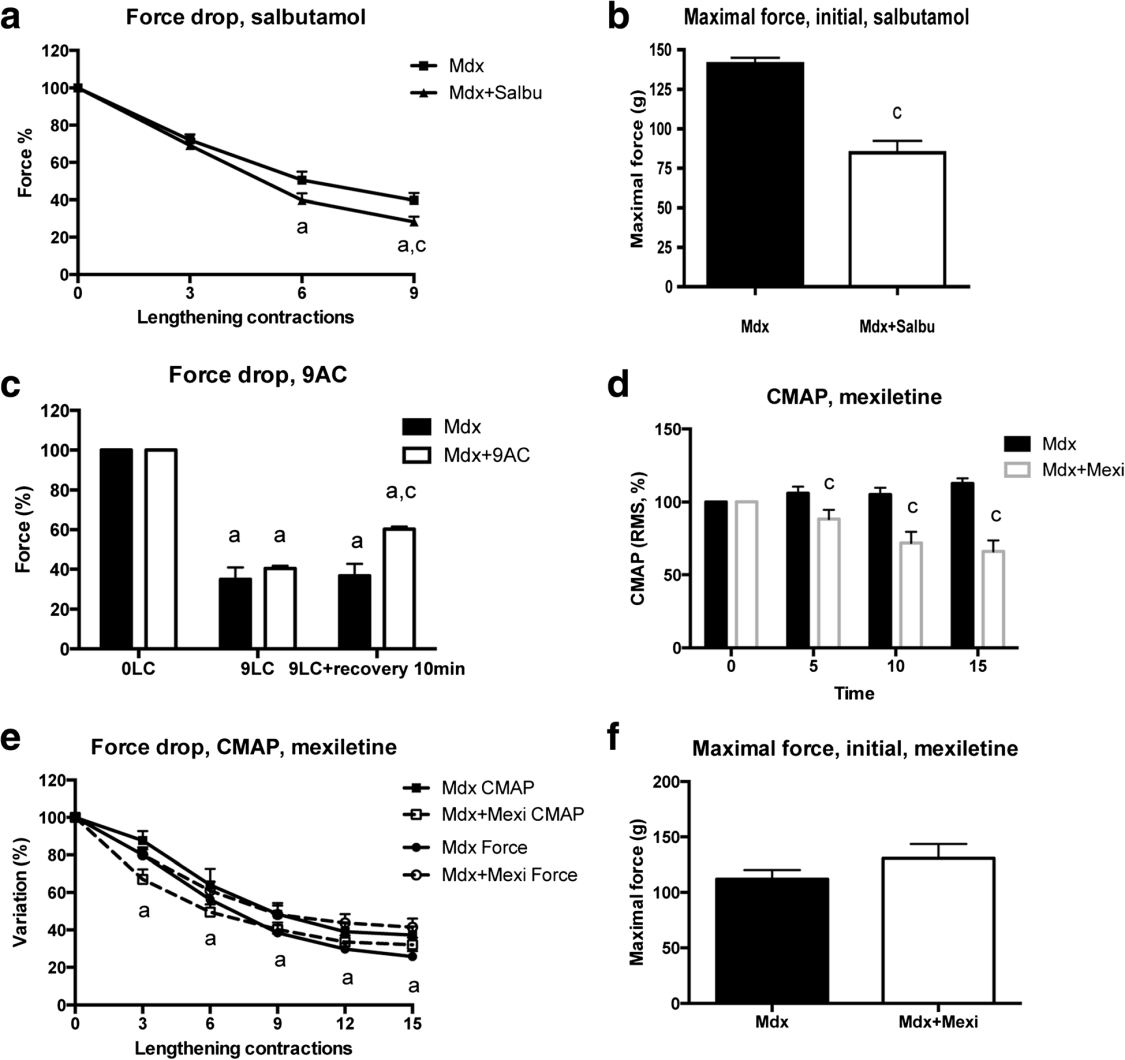
Effect of pharmacological modulation of muscle excitability (salbutamol, 9AC, mexiletine) in the TA muscle from mdx mice. **a** Force drop following lengthening contractions in mdx mice treated with salbutamol. **b** Maximal force before lengthening contractions (initial) in mdx mice treated with salbutamol. **c** Force drop following nine lengthening contractions in mdx mice treated with anthracene-9-carboxylic acid (*9AC*). **d** CMAP (RMS) during 125 Hz stimulation in mdx mice treated with mexiletine, before lengthening contractions. **e** Force and CMAP (RMS) drops following lengthening contractions in mdx mice treated with mexiletine. **f** Maximal force before lengthening contractions in mdx mice treated with mexiletine. *CMAP* compound muscle action potential, *Mdx + Salbu* mdx mice treated with salbutamol, *Mdx + 9AC* mdx mice treated with 9AC, *Mdx + Mexi* mdx mice treated with mexiletine, *Mdx LC* lengthening contractions in mdx mice, *a* significantly different from before lengthening contractions (*p* < 0.05), *c* significant difference with non-treated mice (*p* < 0.05). *n* = 6–15 per group

With the aim to increase muscle excitability, mdx mice were treated, before the lengthening contractions, with salbutamol that activates the Na^+^,K^+^ pump [32]. A recent study suggests that the Na^+^,K^+^ pump is depressed in mdx mice at the basal state, i.e., before lengthening contractions [33]. We found that salbutamol administration (2 mg/kg, ip) did not reduce the force drop following lengthening contractions (Fig. 4a), even though the initial maximal force (before lengthening contractions) was reduced by salbutamol (Fig. 4b) (*p* < 0.05). Mdx mice were then treated after the ninth lengthening contraction, with anthracene-9-carboxylic acid (9AC) (30 mg/kg, ip) that increases muscle excitability via an inhibition of the chloride channel [34]. Mdx mice were treated after (and not before) the lengthening contractions, to avoid complications due to 9AC-induced slowing of the relaxation during lengthening contractions. We found that 9AC treatment improved the maximal force 10 min after the last lengthening contraction in mdx mice (Fig. 4c) (*p* < 0.05). Finally, mdx mice were treated before the lengthening contractions with mexiletine (40 mg/kg, ip), a sodium channel blocker, that reduced muscle excitability [34]. We confirmed that mexiletine decreased CMAP in mdx mice before lengthening contractions (Fig. 4d) (*p* < 0.05). Since mexiletine also induces a tetanic fade (decrease in force within a train of stimulation) with the usual 125-Hz stimulation, we performed 75 Hz stimulation that did not produce such a phenomenon. We found that mexiletine treatment had no effect on either force or CMAP—during lengthening contractions (Fig. 4d, e), suggesting that alteration in sodium channels does not contribute to the reduced excitability following lengthening contractions. It should be noted that mexiletine did not alter initial maximal force (before lengthening contractions) (Fig. 4f). Together, these results may suggest that the reduced excitability following lengthening contractions results, at least partly, from an alteration in chloride channels but not in either the Na^+^,K^+^ pump or sodium channels.

### Is the excitation-contraction uncoupled?

**Fig. 5:**
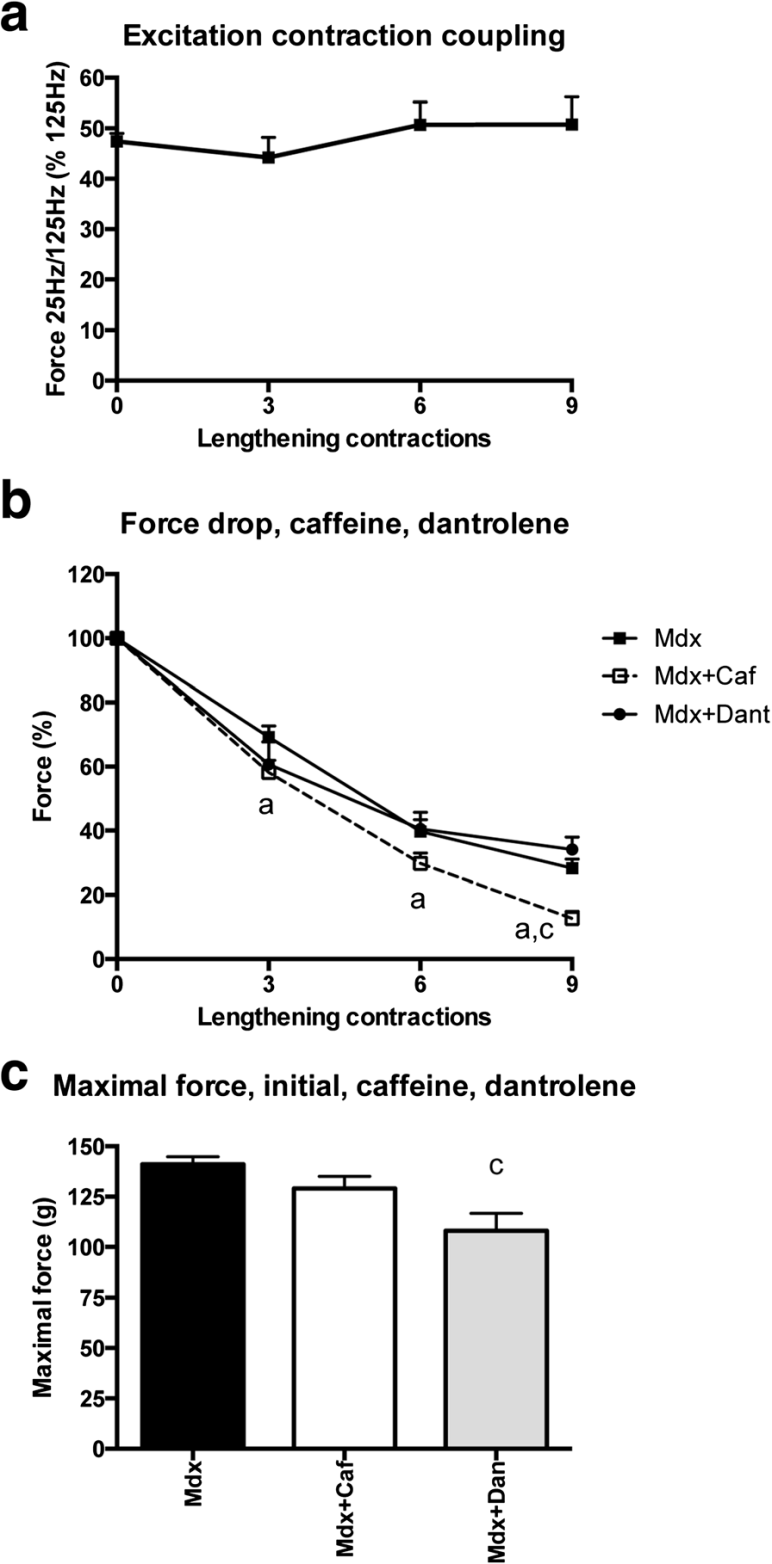
Excitation-contraction coupling (25 Hz/125 Hz force ratio) following lengthening contractions and effect of pharmacological modulation of ryanodine receptor (caffeine, dantrolene) in the TA muscle from mdx mice. **a** Ratio of 25 and 125 Hz forces following lengthening contractions in mdx mice, used as an index of excitation-contraction coupling. **b** Force drop following lengthening contractions in the TA muscle from mdx mice treated with caffeine or dantrolene, i.e., modulators of ryanodine receptor. **c** Maximal force before lengthening contractions (initial) in the TA muscle of mdx mice treated with caffeine or dantrolene. *Mdx + Caf* mdx mice treated with caffeine, *Mdx + Dant* mdx mice treated with dantrolene, *a* significantly different from before lengthening contractions (*p* < 0.05), *c* significant difference with non-treated mice (*p* < 0.05). *n* = 6–10 per group

We then tested the possibility that a defect in excitation-contraction coupling also contributes to the force drop in mdx mice. A decrease in the ratio of the force at low frequency (25 Hz) to the force at high-frequency (125 Hz) stimulation following lengthening contractions was used as a marker of excitation-contraction uncoupling [35, 36]. At low frequency, insufficient calcium would be released (resulting in reduced submaximal force) that could be overcome by high-frequency stimulation (resulting in near normal maximal force). Figure 5a showed that the 25 Hz/125 Hz force ratio did not decrease following lengthening contractions in mdx mice. These results suggest that excitation-contraction uncoupling did not contribute to the force drop following lengthening contractions in mdx mice. However, it remains to be confirmed if this ratio can be applicable to test excitation-contraction coupling following lengthening contractions in mdx muscle.

### Is the calcium release also limited because of RyR alteration?

To determine whether the force drop following lengthening contractions was also increased by further ryanodide receptor (RyR) dysfunction [37, 38], we treated mdx mice with caffeine or dantrolene, two pharmacological agents known to increase or reduce calcium release by modulating RyR functions [35, 39]. Our hypotheses were that caffeine or dantrolene would increase or decrease the calcium leak related to RyR dysfunction, thus the force drop following lengthening contractions. The results shown that caffeine (8 mg/kg, ip) increased the force drop following lengthening contractions in mdx mice (Fig. 5b). However, dantrolene (15 mg/kg, ip) did not reduce it (Fig. 5b). It should be noted that dantrolene reduced maximal force before lengthening contraction (Fig. 5c). These results suggest that a further worsening of RyR dysfunction following lengthening contractions did not contribute to the force drop in mdx mice since dantrolene did not improve it.

### Is the contractile apparatus preserved?

**Fig. 6:**
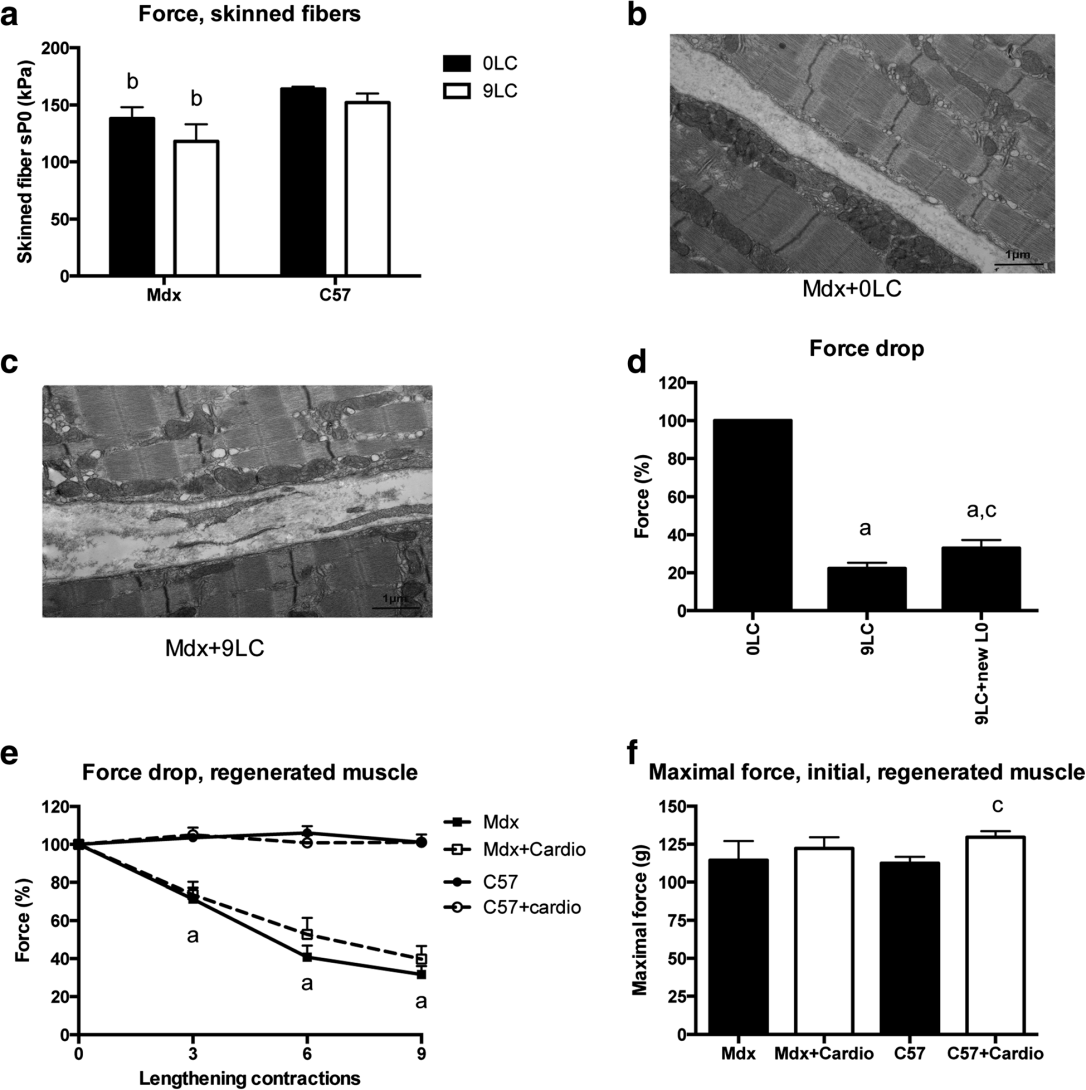
Myofribrillar function, muscle fiber ultrastructure, and optimal muscle length following lengthening contractions in the TA muscle from mdx mice and effect of prior cardiotoxin injection. **a** Calcium maximally activated force of skinned muscle fibers (myofibrillar function) after nine lengthening contractions. **b**, **c** Representative images of electron microscopy from mdx mouse illustrating the absence of morphological alterations following nine lengthening contractions (**c**) as compared to before lengthening contractions (**b**). **d** Force drop following nine lengthening contractions in mdx mice, muscles were stretched to try to obtain a new optimal length (L0). **e** Force drop following lengthening contractions in the cardiotoxin-treated (freshly regenerated) mdx muscle. C57 mice were also shown. **f** Maximal force before lengthening contractions (initial) in mdx muscle treated with cardiotoxin. C57 mice were also shown. *0LC* before lengthening contractions, *9LC* after nine lengthening contractions, *9LC + newL0* muscles were stretched to try to obtain a new optimal length (L0) after nine lengthening contractions, *Cardio* muscle injected with cardiotoxin 3 weeks before, *a* significantly different from before lengthening contractions (*p* < 0.05), *b* significant difference between strains (*p* < 0.05), *c* significantly different from 9LC (*p* < 0.05), *d* significantly different from C57 (*p* < 0.05). *n* ≥ 20 per group for **a**; *n* = 7–10 per group for **d**–**f**

We then tested the possibility that lengthening contractions altered myofibrillar function. Immediately following in situ the nine lengthening contractions, skinned fibers were prepared from the TA muscle and maximal activated calcium force was measured in both mdx and C57 mice. We found no reduction in maximal activated calcium force following the nine lengthening contractions in both mdx- and C57-skinned fibers expressing the types IIx and IIb MHC isoforms (Fig. 6a) (*p* > 0.05), indicating that myofibrillar dysfunction did not contribute to the force drop following lengthening contractions in mdx mice.

Accordingly, we observed that lengthening contractions induced no major change in sarcomere ultrastructure, using electron microscopy (Fig. 6b, c). In line with previous studies [40], we did find morphological abnormalities in some TA muscle fibers of mdx mice before lengthening contractions, such as enlarged SR cisternae, focal Z-line absence or streaming, degenerating fibers, and central nuclei (data not shown). However, a thorough comparison of the contralateral muscle fixed immediately after the nine lengthening contractions did not reveal any specific additional lesions, such as sarcolemmal ruptures, sarcomere tearing, thus arguing against any structural injuries directly linked to lengthening contractions in mdx mice (Fig. 6c).

In agreement with these previous findings, we found no indication of reduced myofilament overlapping, i.e., disrupted sarcomeres, which has been proposed to be responsible for the force drop following lengthening contractions in healthy mice. The popping-sarcomere hypothesis is based on the proposal that during muscle lengthening, the length change will be taken up by the weakest sarcomeres, resulting in no myofilament overlap in the latter ones [41]. At the end of the lengthening, these overstretched sarcomeres do not re-interdigitate, and thus a shift in muscle optimal length for maximal force production (L0) would occur. To test this mechanism, we determined whether muscle recovered maximal force following the nine lengthening contractions when an attempt was made to reach a possible new L0. As showed in Fig. 6d, maximal force was only slightly improved after this procedure, indicating that the presence of overstretched sarcomeres/sarcomere disruption is not the major explanation for the force drop in mdx mice.

### Are freshly regenerated fibers more fragile?

To test the possibility that it is the presence of freshly regenerated fibers but not the dystrophin deficiency per se that causes the susceptibility to lengthening contraction-induced muscle damage, cardiotoxin (10 μM, 50 μl), a myotoxic agent, was injected into TA muscles from mdx mice as described [42]. We found that the force drop following lengthening contractions was not increased by cardiotoxin injection in mdx and C57 mice (Fig. 6e) (*p* > 0.05), at a time (21-day postinjection) where the regenerating muscle have recovered its maximal force production (Fig. 6f). This result indicated that recent muscle degeneration/regeneration was not the cause of the susceptibility to lengthening contraction-induced injury in the mdx mice.

### Does dystrophin restoration by exon skipping improve both force drop and muscle excitability?

**Fig. 7:**
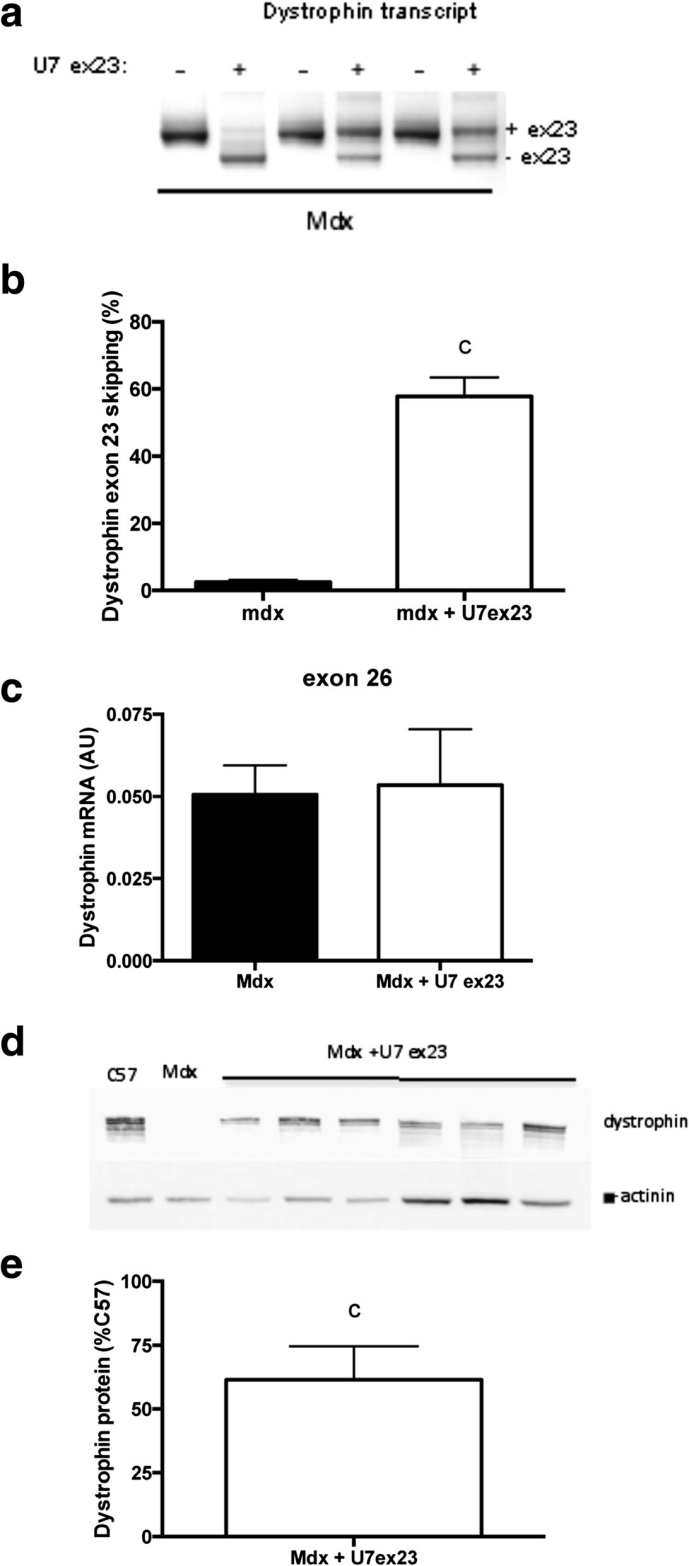
Partial restoration of dystrophin expression in the TA muscle from mdx mice injected with AAV1-U7ex23. **a** RT-PCR analysis of *Dmd* exon 23 skipping in mdx mice injected with AAV1-U7ex23. **b** Quantification of *Dmd* exon 23 skipping in mdx mice injected with AAV1-U7ex23, **c** Dystrophin total mRNA level in mdx mice injected with AAV1-U7ex23. **d** Western blot analysis of dystrophin protein expression in mdx mice injected with AAV1-U7ex23, **e** Quantification of dystrophin protein expression in mdx mice injected with AAV1 U7ex23. *U7-ex23* AAV1-U7ex23, *Mdx* mdx muscle injected with saline, *Mdx + U7ex23* mdx muscle injected with AAV1-U7ex23, *c* significant difference with non-injected muscle (*p* < 0.05). *n* = 6–10 per group

In order to answer this question, we analyzed the effect of the restoration of dystrophin protein expression on force drop and muscle excitability following lengthening contractions. For this purpose, we used AAV-U7snRNA-mediated exon skipping of the *Dmd* ex23 previously described in mdx mice [10]. Intramuscular injection of mdx TA muscles with AAV1-U7ex23 resulted, 3 weeks later, in a partial skipping of exon 23 of *Dmd* (Fig. 7a, b) (*p* < 0.05), without affecting the total level of dystrophin mRNA (Fig. 7c). A partial restoration of dystrophin protein expression was confirmed by Western blot (Fig. 7d, e).

**Fig. 8:**
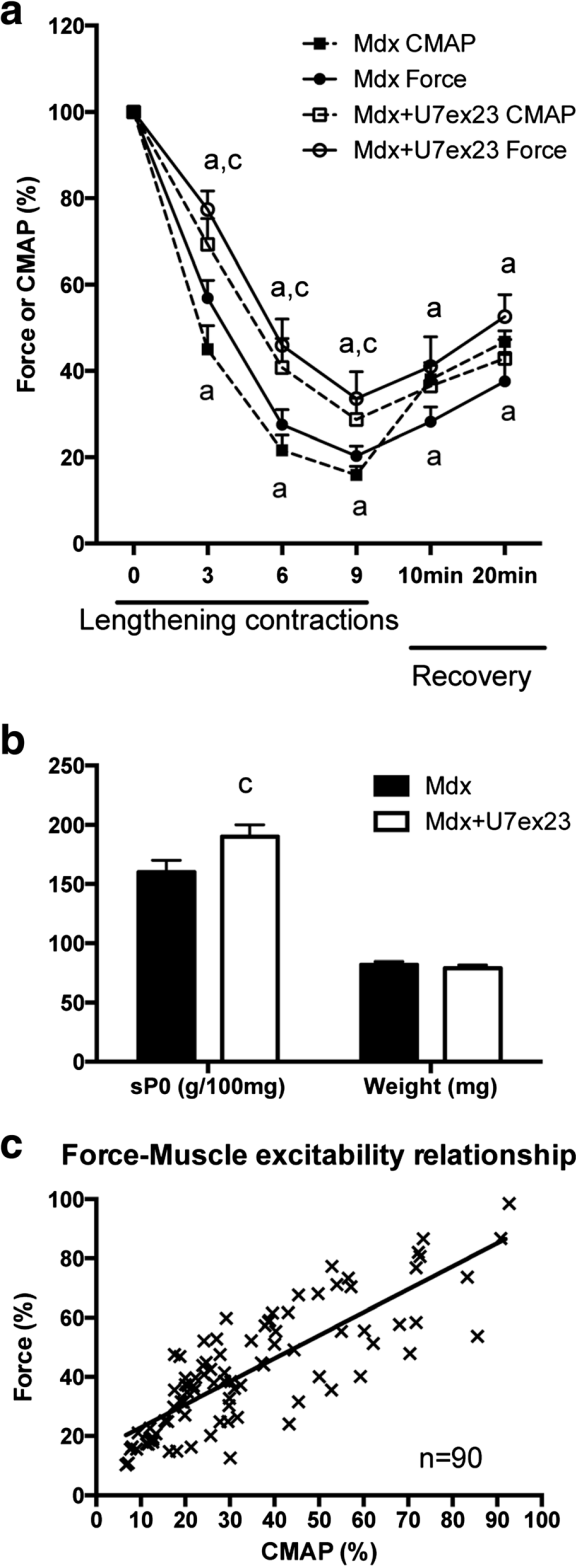
Effect of partial dystrophin restoration in the TA muscle from mdx mice. **a** Force and CMAP (RMS) drops following lengthening contractions in mdx mice injected with AAV1-U7ex23. **b** Specific maximal force and muscle weight before lengthening contractions in mdx mice injected with AAV1-U7ex23. **c** Force and CMAP relationship during lengthening contractions and 20 min recovery in mdx and mdx mice injected with AAV1-U7ex23. *U7-ex23* AAV1-U7ex23, *Mdx* mdx muscle injected with saline, *Mdx + U7ex23* mdx muscle injected with AAV1-U7ex23, *a* significantly different from before lengthening contractions (*p* < 0.05), *c* significant difference with non-injected muscle (*p* < 0.05). *n* = 8–13 per group

We found that 3 weeks after AAV1-U7ex23 injection, the force drop following lengthening contractions was reduced (Fig. 8a) (*p* < 0.05), and specific maximal force was also increased by 19 % (Fig. 8b) (*p* < 0.05), confirming our previous studies [12, 43]. Interestingly, the decrease in CMAP (RMS) following lengthening contractions was also reduced by AAV1-U7ex23 (Fig. 8a) (*p* < 0.05). Another novel result from this experiment was that the changes in force were closely related to those of CMAP during both the lengthening contractions and recovery in both mdx and mdx mice injected with AAV1-U7ex23 (Fig. 8c), with a good correlation between CMAP and absolute maximal force (*r*^2^ = 0.66) (*p* < 0.0001). Together, these results indicate that the beneficial effect of restoration of dystrophin expression on the force drop following lengthening contractions in mdx mice was mediated by the prevention of the CMAP impairment, i.e., reduced muscle excitability.

## Discussion

### The force drop in mdx mice is mainly due to reduced muscle excitability

The present study reveals for the first time that lengthening contractions induced a loss of TA muscle excitability in mdx mice, independently from major neuromuscular transmission failure. Firstly, we found that CMAP was reduced following lengthening contractions in mdx mice, in agreement with a previous study [18]. Secondly, we reported that the voltage needed to elicit 50 % of maximal force increased when the mdx muscle was directly stimulated without the need of neuromuscular transmission, indicating that there was an increased threshold for action potential generation following lengthening contractions. Thirdly, we found that the reduced CMAP following lengthening contractions did not result from major neuromuscular transmission failure in mdx, despite subtle morphological derangements of the neuromuscular junctions.

Importantly, we provided evidence that reduced muscle excitability contributes to the immediate force drop following lengthening contractions in mdx mice. Firstly, we found a strong relationship between CMAP and force following lengthening contractions and recovery in mdx mice. Secondly, the force drop following lengthening contraction can be mimicked by an experimental reduction in muscle excitation, using a neuromuscular transmission blocker (tubocurarine). Thirdly, improving muscle excitability (via increased dystrophin expression) following lengthening contractions and the recovery also reduces the force drop. This mechanism of force drop is also plausible for C57 mice since we found that a marked force drop following lengthening contractions in C57 mice was associated with a reduced CMAP (Fig. 3e).

Another major and novel finding of the present study was that the impaired muscle excitability not only contributes to the force drop following lengthening contractions but it is the major contributor. In fact, there was no major neuromuscular transmission failure, and neither excitation-contraction coupling nor myofibrillar function was altered following lengthening contractions. However, it is not impossible that a different muscle studied and/or protocol of lengthening contractions can also alter myofibrillar function [20] and/or neuromuscular transmission [16]. Mild injury may primarily impact excitability and be rapidly reversible (as in the present study) while more severe injury may result in additional, more slowly reversible effects (including neuromuscular transmission failure, reduced myofibrillar function) [16, 20].

Possible changes in K^+^, Na^+^, Cl^−^, and calcium transarcolemmal gradients following lengthening contractions could theorically explain the reduced muscle excitability in mdx mice, i.e., generation/propagation of action potentials in response to stimulation. As one possibility, lengthening contractions could cause micro-tears in the mdx muscle due to the lack of dystrophin, which leads to membrane potential depolarization and inactivation of Na^+^ channels. However, our results concerning the effects of different channels and pump modulators (mexiletine, salbutamol) suggest that Na^+^ channel and Na^+^,K^+^ pump are not involved in the membrane dysfunction leading to reduced excitability following lengthening contractions. At the basal state (before the lengthening contractions), no clear alteration in the electrophysiology of these sarcolemnal channels [44] supports the notion that their aggravated impairment by lengthening contractions can play a role in the reduced muscle excitability. In contrast, we found that 9AC, a Cl^−^ channel inhibitor increasing excitability, reduced the force drop, suggesting that lengthening contractions could further increase chloride conductance in mdx mice. It was previously reported that chloride conductance is already increased in mdx mice at the basal state [45]. These results suggest that Cl^−^ channel dysfunction could contribute to the reduced excitability following lengthening contractions in mdx mice. However, the only way of determining if Cl^−^ channels are affected is by measuring their current before and after the lengthening contractions. Clearly, future electrophysiological studies are needed to explore the dysfunction of membrane channels in mdx mice.

Since the force drop was marked and we found no other major alterations than reduced muscle excitability, it is very likely that a great number of unexcited TA muscle fibers do not contribute to muscle force production following lengthening contractions in mdx mice. In line, a recent study [18] reported an increased number of depolarized muscle fibers following lengthening contractions in mdx mice, fibers that are very likely not excitable. It remains to be determined whether the mechanisms responsible for the reduced muscle excitability are related to sarcolemmal damage following lengthening contractions as shown by the presence within a few fibers of impermeant dyes [5, 6] and the presence of 12 % malformed fibers exhibiting alterations in action potential kinetics at the basal state [46] in mdx mice.

### The force drop is related to dystrophin

The force drop following lengthening contractions was not increased by prior myotoxic agent injection in mdx mice, indicating that the greater susceptibility to contraction induced injury is not explained by the presence of freshly regenerated dystrophic fibers that could be more fragile, i.e., susceptible to contraction-induced injury. The greater force drop is also not a hallmark of muscles affected by neuromuscular disorders. Indeed, a greater force drop following lengthening contractions is also observed in alpha-sarcoglycan null mice [20] but not in collagen 6A1 null mice, whereas the force drop was reduced in desmin null mice [47]. Together, these findings emphasized the specific role of dystrophin and dystrophin-associated complex in preventing the force drop following lengthening contractions. This notion is also supported by the preclinic therapies based on dystrophin restoration since increased dystrophin expression reduced the force drop following lengthening contractions in mdx mice (see below).

### Dystrophin-based therapy improves muscle excitability

It is thought that the greater susceptibility to contraction-induced damage initiates repeated degeneration/regeneration cycles leading to muscle weakness and wasting in DMD. The greater immediate force drop following lengthening contractions is also an important tool for testing preclinic dystrophin-based therapies for DMD: exon skipping therapy [9–11], microdystrophin therapy [8, 13], dual dystrophin/myostatin therapy [12, 43], dual dystrophin/follistatin therapy [14], and dual dystrophin/nNOS therapy [15]. It has been well established that these dystrophin-based therapies improves the immediate force drop following lengthening contractions in mdx mice [8–15].

In the present study, we demonstrated for the first time that exon skipping-based dystrophin restoration (via AAV1-U7ex23) mediates its beneficial effect by preventing the large reduction in muscle excitability in mdx mice, at least in the TA muscle. Thus, it is very likely that the other preclinic interventions also improve muscle action potential generation/conduction following lengthening contractions, in particular antisense oligonucleotide-mediated splice modification, currently one of the most promising therapeutic strategies for DMD [9, 11]. Moreover, it is possible that dystrophin restoration also contributes to the reduced muscle weakness by improving muscle excitability at the basal state (before lengthening contractions) since specific maximal was 19 % increased by AAV1-U7ex23.

## Conclusions

Our findings revealed that (i) dystrophin is needed to maintain muscle excitability following lengthening contraction and (ii) the reduced muscle excitability largely contributes to the greater immediate force drop following lengthening contractions in the TA muscle from mdx mice. Importantly, we evidenced that dystrophin-based therapy in mdx mice reduces this force drop following lengthening contractions via improved muscle excitability. Thus, the present study provides new insights that are relevant not only for DMD etiology but also for treatment. To enhance efficacy of dystrophin-based therapies, innovative means are needed to help to maintain muscle excitability following lengthening contractions. This notion is important since muscle inexcitability following high-force contractions causes temporary muscle weakness and may be irreversible by contributing to muscle wasting in the long term (such as denervation or unloading).

## References

[1] Allen DG, Gervasio OL, Yeung EW, Whitehead NP (2010). Calcium and the damage pathways in muscular dystrophy. Can J Physiol Pharmacol.

[2] Gumerson JD, Michele DE (2011). The dystrophin-glycoprotein complex in the prevention of muscle damage. J Biomed Biotechnol.

[3] Brooks SV (1998). Rapid recovery following contraction-induced injury to in situ skeletal muscles in mdx mice. J Muscle Res Cell Motil.

[4] Head SI, Williams DA, Stephenson DG (1992). Abnormalities in structure and function of limb skeletal muscle fibres of dystrophic mdx mice. Proc Biol Sci.

[5] Moens P, Baatsen PH, Marechal G (1993). Increased susceptibility of EDL muscles from mdx mice to damage induced by contractions with stretch. J Muscle Res Cell Motil.

[6] Petrof BJ, Shrager JB, Stedman HH, Kelly AM, Sweeney HL (1993). Dystrophin protects the sarcolemma from stresses developed during muscle contraction. Proc Natl Acad Sci USA.

[7] Lynch GS (2004). Role of contraction-induced injury in the mechanisms of muscle damage in muscular dystrophy. Clin Exp Pharmacol Physiol.

[8] Deconinck N, Ragot T, Marechal G, Perricaudet M, Gillis JM (1996). Functional protection of dystrophic mouse (mdx) muscles after adenovirus-mediated transfer of a dystrophin minigene. Proc Natl Acad Sci USA.

[9] Godfrey C (2015). How much dystrophin is enough: the physiological consequences of different levels of dystrophin in the mdx mouse. Hum Mol Genet.

[10] Goyenvalle A (2004). Rescue of dystrophic muscle through U7 snRNA-mediated exon skipping. Science.

[11] Goyenvalle A (2015). Functional correction in mouse models of muscular dystrophy using exon-skipping tricyclo-DNA oligomers. Nat Med.

[12] Hoogaars W (2012). Combined effect of AAV-U7-induced dystrophin exon skipping and soluble activin type IIB receptor in mdx mice. Hum Gene Ther.

[13] Koo T (2011). Delivery of AAV2/9-microdystrophin genes incorporating helix 1 of the coiled-coil motif in the C-terminal domain of dystrophin improves muscle pathology and restores the level of alpha1-syntrophin and alpha-dystrobrevin in skeletal muscles of mdx mice. Hum Gene Ther.

[14] Rodino-Klapac LR (2013). Micro-dystrophin and follistatin co-delivery restores muscle function in aged DMD model. Hum Mol Genet.

[15] Zhang Y (2013). Dual AAV therapy ameliorates exercise-induced muscle injury and functional ischemia in murine models of Duchenne muscular dystrophy. Hum Mol Genet.

[16] Pratt SJ (2013). Effects of in vivo injury on the neuromuscular junction in healthy and dystrophic muscles. J Physiol.

[17] Pratt SJ (2015). Recovery of altered neuromuscular junction morphology and muscle function in mdx mice after injury. Cell Mol Life Sci.

[18] Call JA, Warren GL, Verma M, Lowe DA (2013). Acute failure of action potential conduction in mdx muscle reveals new mechanism of contraction-induced force loss. J Physiol.

[19] Blaauw B (2008). Akt activation prevents the force drop induced by eccentric contractions in dystrophin-deficient skeletal muscle. Hum Mol Genet.

[20] Blaauw B (2010). Eccentric contractions lead to myofibrillar dysfunction in muscular dystrophy. J Appl Physiol.

[21] Ferry A (2015). Effect of voluntary physical activity initiated at age 7 months on skeletal hindlimb and cardiac muscle function in mdx mice of both genders. Muscle Nerve.

[22] Hourde C (2013). Protective effect of female gender-related factors on muscle force-generating capacity and fragility in the dystrophic mdx mouse. Muscle Nerve.

[23] Ferry A (2014). Advances in the understanding of skeletal muscle weakness in murine models of diseases affecting nerve-evoked muscle activity, motor neurons, synapses and myofibers. Neuromuscul Disord.

[24] Morsch M, Reddel SW, Ghazanfari N, Toyka KV, Phillips WD (2012). Muscle specific kinase autoantibodies cause synaptic failure through progressive wastage of postsynaptic acetylcholine receptors. Exp Neurol.

[25] Joanne P (2012). Impaired adaptive response to mechanical overloading in dystrophic skeletal muscle. PLoS One.

[26] Frontera WR, Larsson L (1997). Contractile studies of single human skeletal muscle fibers: a comparison of different muscles, permeabilization procedures, and storage techniques. Muscle Nerve.

[27] Messéant J (2015). MuSK frizzled-like domain is critical for mammalian neuromuscular junction formation and maintenance. J Neurosci Off J Soc Neurosci.

[28] Courchesne SL, Pazyra-Murphy MF, Lee DJ, Segal RA (2011). Neuromuscular junction defects in mice with mutation of dynein heavy chain 1. PLoS One.

[29] Goyenvalle A (2012). Rescue of severely affected dystrophin/utrophin-deficient mice through scAAV-U7snRNA-mediated exon skipping. Hum Mol Genet.

[30] Cairns SP, Taberner AJ, Loiselle DS (2009). Changes of surface and t-tubular membrane excitability during fatigue with repeated tetani in isolated mouse fast- and slow-twitch muscle. J Appl Physiol.

[31] Mouisel E (2006). Outcome of acetylcholinesterase deficiency for neuromuscular functioning. Neurosci Res.

[32] Clausen T, Nielsen OB, Clausen JD, Pedersen TH, Hayward LJ (2011). Na+,K+-pump stimulation improves contractility in isolated muscles of mice with hyperkalemic periodic paralysis. J Gen Physiol.

[33] Miles MT, Cottey E, Cottey A, Stefanski C, Carlson CG (2011). Reduced resting potentials in dystrophic (mdx) muscle fibers are secondary to NF-kappaB-dependent negative modulation of ouabain sensitive Na+-K+ pump activity. J Neurol Sci.

[34] Desaphy JF, Carbonara R, Costanza T, Conte Camerino D (2014). Preclinical evaluation of marketed sodium channel blockers in a rat model of myotonia discloses promising antimyotonic drugs. Exp Neurol.

[35] Balnave CD, Allen DG (1995). Intracellular calcium and force in single mouse muscle fibres following repeated contractions with stretch. J Physiol.

[36] Willems ME, Stauber WT (2009). The effect of number of lengthening contractions on rat isometric force production at different frequencies of nerve stimulation. Acta Physiol.

[37] Bellinger AM (2009). Hypernitrosylated ryanodine receptor calcium release channels are leaky in dystrophic muscle. Nat Med.

[38] Li D, Yue Y, Lai Y, Hakim CH, Duan D (2011). Nitrosative stress elicited by nNOSmicro delocalization inhibits muscle force in dystrophin-null mice. J Pathol.

[39] Warren GL (1993). Excitation failure in eccentric contraction-induced injury of mouse soleus muscle. J Physiol.

[40] Torres LF, Duchen LW (1987). The mutant mdx: inherited myopathy in the mouse. Morphological studies of nerves, muscles and end-plates. Brain.

[41] Proske U, Morgan DL (2001). Muscle damage from eccentric exercise: mechanism, mechanical signs, adaptation and clinical applications. J Physiol.

[42] Mouisel E, Vignaud A, Hourdé C, Butler-Browne G, Ferry A (2010). Muscle weakness and atrophy are associated with decreased regenerative capacity and changes in mTOR signaling in skeletal muscles of venerable (18-24-month-old) dystrophic mdx mice. Muscle Nerve.

[43] Dumonceaux J (2010). Combination of myostatin pathway interference and dystrophin rescue enhances tetanic and specific force in dystrophic mdx mice. Mol Ther.

[44] Allard B (2006). Sarcolemmal ion channels in dystrophin-deficient skeletal muscle fibres. J Muscle Res Cell Motil.

[45] De Luca A (2003). Enhanced dystrophic progression in mdx mice by exercise and beneficial effects of taurine and insulin-like growth factor-1. J Pharmacol Exp Ther.

[46] Hernandez-Ochoa EO, Pratt SJ, Garcia-Pelagio KP, Schneider MF, Lovering RM (2015). Disruption of action potential and calcium signaling properties in malformed myofibers from dystrophin-deficient mice. Physiol Rep.

[47] Sam M (2000). Desmin knockout muscles generate lower stress and are less vulnerable to injury compared with wild-type muscles. Am J Physiol Cell Physiol.

